# Comparing Survival of Perihilar Cholangiocarcinoma After R1 Resection Versus Palliative Chemotherapy for Unresected Localized Disease

**DOI:** 10.1245/s10434-024-15582-5

**Published:** 2024-06-19

**Authors:** Anne-Marleen van Keulen, Stefan Buettner, Pim B. Olthof, Heinz-Josef Klümpen, Joris I. Erdmann, Laura Izquierdo-Sanchez, Jesus M. Banales, Benjamin Goeppert, Stephanie Roessler, Krzysztof Zieniewicz, Angela Lamarca, Juan W. Valle, Adelaida La Casta, Frederik J. H. Hoogwater, Matteo Donadon, Alexander Scheiter, Marco Marzioni, Jorge Adeva, Edita Kiudeliene, Jesús María Urman Fernández, Gianpaolo Vidili, Tudor Mocan, Luca Fabris, Marcin Krawczyk, Trine Folseraas, Cristina Dopazo, Olivier Detry, Theodor Voiosu, Viorel Scripcariu, Francesca Biancaniello, Chiara Braconi, Rocio I. R. Macias, Bas Groot Koerkamp

**Affiliations:** 1 Department of Surgery, Erasmus MC Cancer Institute, Rotterdam, The Netherlands; 2 Department of Medical Oncology, Cancer Center Amsterdam, Amsterdam UMC, University of Amsterdam, Amsterdam, The Netherlands; 3 Department of Surgery, Cancer Center Amsterdam, Amsterdam UMC, University of Amsterdam, Amsterdam, The Netherlands; 4 Department of Liver and Gastrointestinal Diseases, Biodonostia Health Research Institute, Donostia University Hospital, University of the Basque Country (UPV/EHU), San Sebastian, Spain; 5 National Institute for the Study of Liver and Gastrointestinal Diseases, Instituto de Salud Carlos III” (ISCIII), CIBERehd, Madrid, Spain; 6 Department of Biochemistry and Genetics, School of Sciences, University of Navarra, Pamplona, Spain; 7 Basque Foundation for Science, Bilbao, Spain; 8 Institute of Pathology and Neuropathology, RKH Klinikum Ludwigsburg, Ludwigsburg, Germany; 9 Institute of Pathology, Kantonsspital Baselland, Liestal, Switzerland; 10 Institute of Pathology, University Hospital Heidelberg, Heidelberg, Germany; 11 Department of General, Transplant and Liver Surgery, Medical University of Warsaw, Warsaw, Poland; 12 Department of Oncology – OncoHealth Institute, Fundación Jiménez Díaz University Hospital, Madrid, Spain; 13 Department of Medical Oncology, The Christie NHS Foundation, Manchester, England; 14 Division of Cancer Sciences, University of Manchester, Manchester, UK; 15 Medical Oncology Department, OSI Donostialdea/Biodonostia, San Sebastián, Spain; 16 University Medical Center Groningen, University of Groningen, Groningen, The Netherlands; 17 Department of Hepatobiliary and General Surgery, IRCCS Humanitas Research Hospital, Rozzano, Milan, Italy; 18 Institute of Pathology, University of Regensburg, Regensburg, Germany; 19 Clinic of Gastroenterology and Hepatology, Universita Politecnica delle Marche, Ancona, Italy; 20 Department of Medical Oncology, Hospital Universitario, 12 de Octubre, Madrid, Spain; 21 Department of Gastroenterology, Lithuanian University of Health Sciences, Kaunas, Lithuania; 22 Servicio de Aparato Digestivo, Complejo Hospitalario de Navarra, Pamplona, Navarra España; 23 Department of Medicine, Surgery and Pharmacy, University of Sassari, Sassari, Italy; 24 Department of Internal Medicine, Day Hospital of the Medical Area, Azienda Ospedaliero Universitaria, AOU, Sassari, Italy; 25 Babeș-Bolyai University - UBB Med Department, Regional Institute of Gastroenterology and Hepatology, Cluj-Napoca, Romania; 26 Department of Molecular Medicine, University of Padua School of Medicine, Padua, Italy; 27 Digestive Disease Section, Yale University School of Medicine, New Haven, CT USA; 28 Laboratory of Metabolic Liver Diseases, Medical University of Warsaw, Warsaw, Poland; 29 Department of Medicine II, Saarland University Medical Center, Homburg, Germany; 30 Section of Gastroenterology and the Norwegian PSC Research Center, Department of Transplantation Medicine, Oslo University Hospital, Oslo, Norway; 31 Department of HPB Surgery and Transplants, Vall d’Hebron Hospital Universitari, Vall d’Hebron Institut de Recerca (VHIR), Vall d’Hebron Barcelona Hospital Campus, Universitat Autónoma de Barcelona, Barcelona, Spain; 32 Department of Abdominal Surgery and Transplantation, CHU Liege, University of Liege, Liege, Belgium; 33 Gastroenterology Department, Faculty of Medicine, Colentina Clinical Hospital, UMF Carol Davila, Bucharest, Romania; 34 University of Medicine and Pharmacy “Gr T Popa”, Regional Institute of Oncology, Iasi, Romania; 35 Department of Translational and Precision Medicine, Sapienza” University of Rome, Rome, Italy; 36 Royal Marsden NHS Trust, London, Surrey, UK; 37 Beatson West of Scotland Cancer Centre, Glasgow, UK; 38 School of Cancer Sciences, University of Glasgow, Glasgow, UK; 39 Experimental Hepatology and Drug Targeting (HEVEPHARM) Group, University of Salamanca, IBSAL, CIBERehd, Salamanca, Spain

## Abstract

**Background:**

Resection of perihilar cholangiocarcinoma (pCCA) is a complex procedure with a high risk of postoperative mortality and early disease recurrence. The objective of this study was to compare patient characteristics and overall survival (OS) between pCCA patients who underwent an R1 resection and patients with localized pCCA who received palliative systemic chemotherapy.

**Methods:**

Patients with a diagnosis of pCCA between 1997–2021 were identified from the European Network for the Study of Cholangiocarcinoma (ENS-CCA) registry. pCCA patients who underwent an R1 resection were compared with patients with localized pCCA (i.e., nonmetastatic) who were ineligible for surgical resection and received palliative systemic chemotherapy. The primary outcome was OS.

**Results:**

Overall, 146 patients in the R1 resection group and 92 patients in the palliative chemotherapy group were included. The palliative chemotherapy group more often underwent biliary drainage (95% vs. 66%, *p* < 0.001) and had more vascular encasement on imaging (70% vs. 49%, *p* = 0.012) and CA 19.9 was more frequently >200 IU/L (64 vs. 45%, *p* = 0.046). Median OS was comparable between both groups (17.1 vs. 16 months, *p* = 0.06). Overall survival at 5 years after diagnosis was 20.0% with R1 resection and 2.2% with chemotherapy. Type of treatment (i.e., R1 resection or palliative chemotherapy) was not an independent predictor of OS (hazard ratio 0.76, 95% confidence interval 0.55–1.07).

**Conclusions:**

Palliative systemic chemotherapy should be considered instead of resection in patients with a high risk of both R1 resection and postoperative mortality.

Perihilar cholangiocarcinoma (pCCA) is a rare disease, with an annual incidence of one to two per 100,000 in Western countries.^1^ The median overall survival (OS) after surgical resection is approximately 30 months, and the 5-year OS approximately 30%.^2–4^ However, only approximately 15% of patients with pCCA undergo a curative intent resection.^5^ The majority of patients with pCCA present with metastatic disease, locally advanced disease, or are unfit to undergo major liver resection. Most experts agree that patients with metastatic (i.e., stage IV) pCCA are unlikely to benefit from a resection.^6^

A resection of pCCA is recommended when a complete (i.e., margin-negative [R0]) resection is likely with an acceptable 90-day postoperative mortality. Approximately one third of patients, however, undergo a histological margin-positive (i.e., R1) resection.^7^ The median OS after an R1 resection is approximately 18 months, and the 5-year OS is approximately 10%.^8–14^ The 90-day mortality after resection for pCCA was approximately 12% in two nationwide series but increased to 25% in patients with multiple risk factors.^4,15,16^ It is not known whether patients with pCCA benefit from an R1 resection compared with palliative systemic chemotherapy.

Cross-sectional imaging is inadequate to determine the biliary extent of the tumor and predict how likely an R0 resection is. Moreover, it often is uncertain on imaging whether vascular abutment requires reconstruction of the hepatic artery and portal vein to obtain a negative margin.^17^ A more extended resection (e.g., extended right hemihepatectomy with vascular reconstruction) is more likely to result in a negative margin but also increases the risk of postoperative mortality.

The alternative to surgical resection of localized pCCA is palliative systemic chemotherapy or best supportive care. The median OS with palliative systemic chemotherapy (the standard of care cisplatin–gemcitabine) for advanced biliary tract cancer was 11.7 months in the ABC-02 trial, albeit including patients with metastatic disease and patients with Eastern Cooperative Oncology Group (ECOG) performance status 2.^18^ Overall survival beyond 3 years is rarely observed after palliative systemic chemotherapy in patients with pCCA. Best supportive care (including palliative biliary drainage) is associated with a median OS of only 5 months.^19^

Starting from these observations, we hypothesized that patients with pCCA who underwent an R1 resection may have a similar OS compared with patients with localized pCCA who were ineligible for surgical resection and who received palliative systemic chemotherapy. The purpose of this retrospective cohort study was to compare patient characteristics and OS between patients with localized pCCA who underwent an R1 resection versus palliative systemic chemotherapy.

## Methods

### ENS-CCA Registry

Patients were selected from the European Network for the Study of Cholangiocarcinoma (ENS-CCA) registry, which is a multicenter, international, collaborative research network that aims to improve the understanding of cholangiocarcinoma (intrahepatic, perihilar, and distal) and to improve patient outcomes.^20,21^ The registry includes consecutive patients diagnosed with pCCA at 26 referral hospitals from 11 European countries (Austria, France, Germany, Italy, Lithuania, Netherlands, Norway, Poland, Romania, Spain, and United Kingdom). Patient data, tumor characteristics, and outcomes of (non-)surgical treatment were included in the registry.

### Included Patients

Patients with a diagnosis of pCCA between 1997–2021 were retrospectively included. Two cohorts of patients were selected. The first cohort included patients who underwent a curative-intent resection for pCCA with one or more positive resection margins (R1) upon histopathologic examination. Patients with R2 resection margins (macroscopic residual disease) were excluded. The second cohort included patients with localized (nonmetastatic) pCCA who were considered ineligible for surgical resection but received palliative systemic chemotherapy. Patients considered ineligible for resection had locally advanced disease on imaging (i.e., liver remnant too small or extensive vascular reconstruction needed) and/or a poor performance status. Patients were excluded in case they underwent liver transplantation or had metastatic (M1) disease on preoperative imaging, at staging laparoscopy, or laparotomy. In accordance with the AJCC 8^th^ edition, extraregional lymph node involvement was considered distant metastatic disease.^22^

### Patient Workup and Management

Workup and perioperative management differed across centers because of the multicenter and retrospective study design. Selected patients were treated with (neo)adjuvant therapy. Neoadjuvant therapy consisted primarily of radiotherapy (3x3.5 Gray), and adjuvant therapy consisted of (radio)chemotherapy with either gemcitabine, cisplatin, capecitabine, 5-fluorouracil, oxaliplatin, or a combination of these agents. Palliative patients with localized pCCA were treated with systemic chemotherapy, which consisted of gemcitabine, cisplatin, capecitabine, oxaliplatin, or a combination of these systemic therapies. Diagnosis of pCCA was confirmed at the histopathological level in all patients of the resection group, whereas patients who did not undergo a resection were diagnosed by brush cytology, biopsy, or high clinical suspicion (clinical presentation, serum tumor biomarkers (i.e., CA19-9 and CEA), and radiological imaging).

### Definition and Outcomes

Pathology records that described the positive (R1) resection margins were considered as incomplete resections, with likely residual cancer cells in the transection surface. Overall survival was calculated from the date of diagnosis (cytologic/histologic confirmation or radiological imaging if pathology was not available) to the date of death or last follow-up.

### Statistical Analysis

Categorical variables were expressed as numbers with percentages and analyzed by using the chi-squared or Fisher’s exact test. Continuous variables were reported as median with interquartile range (IQR) and were tested by using Mann-Whitney *U* tests. Multiple imputations were performed by using the MICE package for R (www.r-project.net). Survival curves were generated by using the Kaplan-Meier method. Differences in survival curves were tested by using the log-rank test. A Cox regression analysis was conducted to determine factors associated with OS in a multivariable model. All variables with *p* ≤ 0.1 were entered into the multivariable analyses by using backward selection.

## RESULTS

### Patient Characteristics

**Fig. 1:**
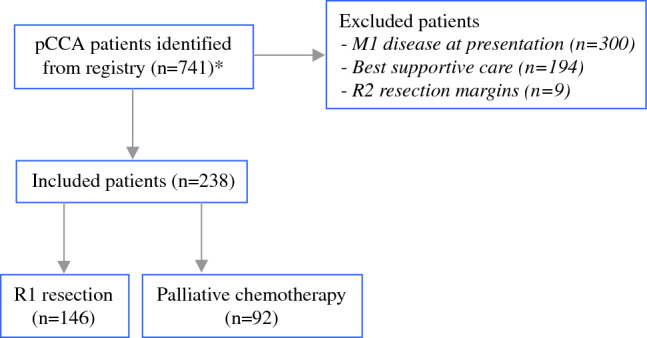
*Patients who underwent an R0 resection or liver transplantation were excluded at preselection

A total of 741 patients with localized pCCA from 25 participating centers in ten European countries were identified in the registry. Patients were excluded if they had metastatic disease at presentation (*n* = 300), an R2 resection (*n* = 9), or received best supportive care (*n* = 194). A flowchart is presented in Fig. 1, and the baseline characteristics of the 238 included patients are shown in Table 1. Patients who underwent a curative-intent resection with R1 resection margins upon histopathologic examination represent the R1 resection group (*n* = 146). Patients with localized pCCA who were ineligible for surgical resection and who received palliative systemic chemotherapy represent the palliative chemotherapy group (*n* = 92). In the palliative group, pathological confirmation of pCCA was obtained in 62 patients (67.4%). Of the patients who underwent a resection, 13 patients (8.9%) were treated with neoadjuvant therapy (*n* = 10 with radiotherapy, *n* = 3 with chemotherapy), and 34 patients (24.3%) received adjuvant systemic chemotherapy. The majority of the included patients was treated in the past 10 years (74.4%).

**Table 1:** Baseline characteristics

		R1 resection (*n* = 146)	Palliative chemotherapy (*n* = 92)	*p*
Sex (%)	Male	78 (53)	52 (57)	0.640
Age (median [IQR])		67.0 [58.0, 72.0]	63.0 [52.8, 70.0]	0.088
BMI (median [IQR])		23.5 [21.2, 26.1]	24.9 [22.2, 29.8]	0.018
PSC (%)		8 (6)	1 (1)	0.122
Liver cirrhosis (%)		2 (1)	1 (1)	0.885
Biliary stent placement (%)		61 (66)	86 (95)	<0.001
ECOG (%)	0	47 (55)	35 (38)	0.093
	1	33 (38)	46 (51)	
	2	5 (6)	10 (11)	
	3	1 (1)	0 (0)	
Vascular encasement on imaging (%)		37 (49)	42 (70)	0.012
Bismuth classification (%)	1	4 (3)	6 (12)	0.235
	2	14 (12)	4 (8)	
	3a	20 (17)	6 (12)	
	3b	19 (16)	7 (14)	
	4	62 (52)	26 (53)	
Tumor differentiation (%)	Well	14 (11)	2 (9)	0.720
	Moderate	79 (61)	12 (55)	
	Poor	36 (28)	8 (36)	
pT stage AJCC8 (%)	T1	2 (2)	*NA*	
	T2	77 (60)	*NA*	
	T3	34 (27)	*NA*	
	T4	15 (12)	*NA*	
pN stage AJCC8 (%)	N0	55 (43)	*NA*	
	N1	59 (46)	*NA*	
	N2	14 (11)	*NA*	
Bilirubin (median [IQR])		19.0 [2.7, 125.0]	4.0 [1.1, 74.0]	0.104
CEA (median [IQR])		3.5 [1.9, 8.6]	3.2 [2.3, 6.2]	0.715
CA 19-9 (median [IQR])		139.4 [37.8, 649.5]	377.0 [89.0, 1011.5]	0.060
CA 19-9 >200		29 (45)	35 (64)	0.046

*CA19-9* carbohydrate antigen 19-9; *CEA* carcinoembryonic antigen

Patients in the palliative chemotherapy group had a higher body mass index (BMI) (24.9 vs. 23.5, *p* = 0.018) and underwent biliary stent placement more frequently prior to treatment (95% vs. 66%, *p* < 0.001). No difference was found in ECOG performance status. Patients who underwent palliative systemic therapy more often had vascular encasement on imaging (70% vs. 49%, *p* = 0.012) and CA 19.9 was more frequently >200 IU/L (64 vs. 45%, *p* = 0.046).

### Overall Survival

**Fig. 2:**
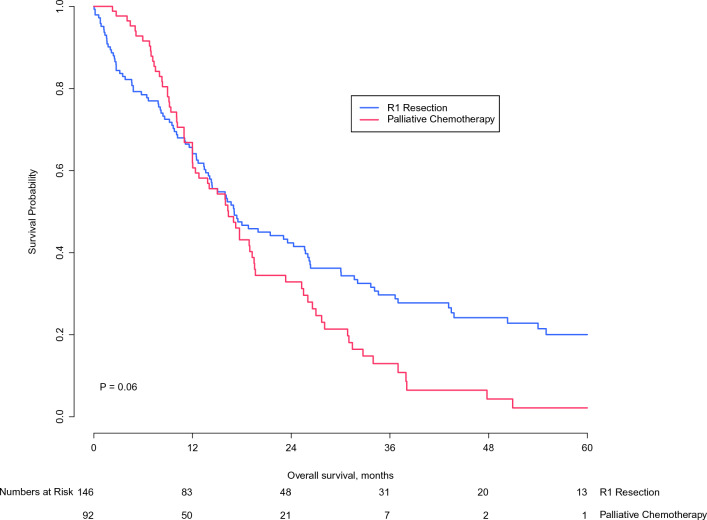
Overall survival of pCCA patients who underwent R1 resection and patients with localized pCCA who received palliative systemic chemotherapy

Sixty-five patients (27.4%) were alive at last follow-up. The median follow-up for patients alive at the last follow-up was 22.3 months for patients in the R1 resection group and 6.4 months for patients in the palliative chemotherapy group. Postoperative mortality at 90 days was 19.9%. Median OS was 17.1 months (95% CI 10.8–23.3) for the R1 resection group and 16.0 months (95% CI 11.4–20.6) for the palliative chemotherapy group (*p* = 0.06; Fig. 2). Estimated survival at 6 months from diagnosis was 78.5% after R1 resection versus 91.6% after palliative systemic chemotherapy. Overall survival for R1 resection versus palliative chemotherapy at 1 year was 64.1% versus 61.9%, at 3 years 29.7% versus 12.9%, and at 5 years 20.0% versus 2.2%.

**Fig. 3:**
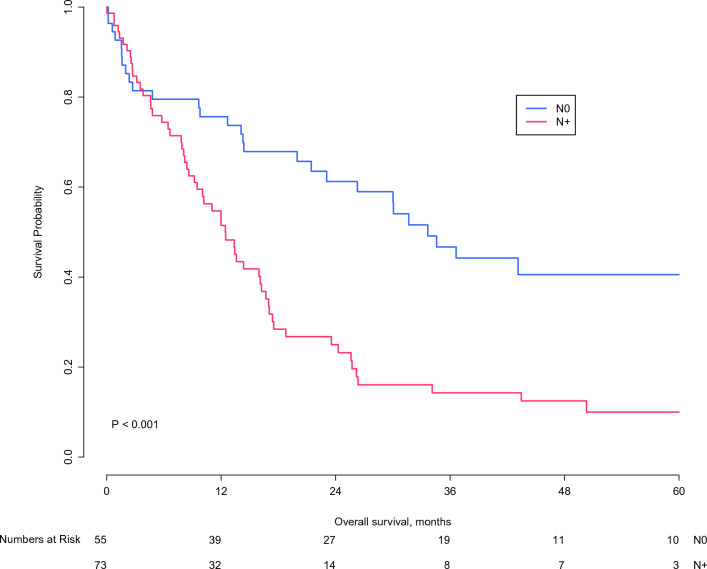
Overall survival of patients who underwent a resection and had negative lymph nodes (N0) compared with positive lymph nodes (N+; N1 or N2)

Within the resection group, a median OS of 12.5 months (95% CI 9.3–15.6) was found for patients with positive lymph nodes (N1/2) compared with 33.7 months (95% CI 25.9–41.4) median OS for negative (N0) lymph nodes (*p* < 0.001; Fig. 3).

Uni- and multivariable analyses are shown in Table 2. Advanced age was an independent poor prognostic factor for all patients (HR 1.02, 95% CI 1.00–1.03). Type of treatment (i.e., R1 resection or palliative chemo) was not an independent predictor of OS (HR 0.76, 95% CI 0.55–1.07).

**Table 2:** Univariable and multivariable analyses for factors associated with OS

	Univariable			Multivariable		
	HR	95% CI	*p*	HR	95% CI	*p*
Male sex	1.31	0.96–1.77	0.086	1.19	0.87–1.63	0.267
Age	1.02	1.00–1.03	0.017	1.02	1.00–1.03	0.035
BMI	1.03	0.99–1.07	0.199			
PSC	0.90	0.44–1.85	0.782			
Liver cirrhosis	3.51	0.86–14.42	0.081	2.23	0.37–13.36	0.359
Biliary drainage	1.23	0.77–1.97	0.383			
ECOG						
Stage 1	Ref	–	–			
Stage 2	1.23	0.86–1.76	0.257			
Stage 3	1.29	0.67–2.47	0.451			
Stage 4	1.05	0.14–7.59	0.963			
Vascular encasement	0.88	0.58–1.32	0.521			
Bismuth						
Stage 1–2	Ref	–	–	Ref	–	–
Stage 3–4	0.62	0.38–1.01	0.053	0.71	0.57–1.08	0.106
Tumor differentiation						
Well-moderate	Ref	–	–			
Poor	1.19	0.79–1.78	0.404			
CEA	1.00	0.99–1.00	0.251			
CA19-9	1.00	1.00–1.00	0.012	1.00	1.00–1.00	0.336
Surgical resection	0.741	0.54–1.01	0.059	0.76	0.55–1.07	0.112

## Discussion

This study compared 146 patients who underwent an R1 resection for pCCA with 92 patients with localized pCCA who received palliative systemic chemotherapy selected from the ENS-CCA registry. Despite more vascular invasion and higher CA 19.9 levels in the palliative systemic chemotherapy group, median OS was comparable between the two groups (17.1 vs. 16.0 months, *p* = 0.06), and type of treatment was not an independent predictor of OS. Estimated survival at 6 months after diagnosis was lower after resection (78.5% vs. 91.6%), mainly due to postoperative mortality. Estimated survival at 5 years after diagnosis, however, was 20.0% after resection and negligible (2.2%) after palliative treatment.

The decision between resection and palliative chemotherapy for patients with localized pCCA can be challenging. Only one third of patients who undergo surgical exploration for pCCA can expect a favorable outcome, defined as an R0 resection without 90-day mortality.^23^ Occult metastatic or locally advanced (i.e., unresectable) disease at exploration is the most common cause of unfavorable outcome. Postoperative mortality is another important factor affecting outcome, with a 90-day mortality in nationwide studies of approximately 10%.^4,15^ Patients with multiple risk factors, such as advanced age, small volume of the liver remnant, and preoperative cholangitis, have a predicted 90-day postoperative mortality that may exceed 25%.^24^.The third cause of unfavorable outcome after resection of pCCA is an R1 resection, which is strongly associated with poor OS.

The long-term survival benefit of resection should clearly outweigh the risk of 90-day mortality. An R1 resection is a well-established poor prognostic factor.^25^ The median OS after an R1 resection is only approximately 18 months and the 5-year OS approximately 10%.^8–14^ In the present study, we found that the median OS for pCCA after R1 resection was similar to palliative chemotherapy. Five-year survival in the resection group, however, was clearly superior at 20.0% (vs. 2.2% in the palliative chemotherapy), at a cost of a 90-day mortality of 19.9%. This presents a difficult trade-off between long-term benefit and short-term harm for patients and their multidisciplinary team.

A prognostic model for OS after resection of pCCA found three independent poor prognostic factors: nodal disease, margin status, and moderate/poor tumor differentiation.^25^ These factors, however, are largely unknown when deciding between surgery and palliative systemic chemotherapy. Cure of pCCA after resection in patients with lymph node-positive disease (N+; N1 or N2) is exceedingly rare.^26^ Within the R1 resection group of the present study, the median OS was only 12.5 months in patients with N+ disease compared with 33.7 months in patients with N0 disease. Prognosis of N+ pCCA is so poor that in the presence of positive regional lymph nodes, resection margin status is no longer associated with OS after resection.^27^ Therefore, we recommend preoperative (with EUS) and intraoperative (with frozen sections) assessment of lymph node status in patients with a high risk of postoperative mortality.

One of the most ambitious goals of surgery for pCCA is to increase the chance of an R0 resection.^7^ Strategies, such as extended hepatectomy or routine portal vein resection, have been proposed to increase the chance of R0 resections.^28^ Vascular resections of the portal vein or hepatic artery may help to achieve R0 resection margins but with a substantial increase in both postoperative morbidity and mortality.^29^ Mizuno et al. from Japan have argued that vascular resections with reconstruction of the portal vein and/or hepatic artery should be performed in patients who often are considered as unresectable by many Western centers.^30^ In-hospital or 90-day mortality was slightly higher in the vascular resection group compared with the no vascular resection group (3.6% vs. 1.2%, *p* = 0.040). The median OS following a vascular resection was shorter (30 months) compared with no vascular resection (61 months) but still longer than the median OS of patients who did not undergo a resection (10 months). Both the postoperative mortality and long-term OS, however, were much more favorable than has been published by any Western center.

Several limitations of the present study should be acknowledged. The retrospective nature has led to selection bias involving the two study cohorts. Patients who underwent a resection differed from those who underwent palliative systemic chemotherapy; on average, the former had less advanced disease and a better performance status. This could partly explain superior 5-year OS after resection compared with palliative systemic chemotherapy. Second, the long study period may have biased results, because both surgical and palliative treatment have evolved over time. In particular, the addition of immunotherapy (durvalumab) in the TOPAZ-1 randomized controlled trial showed improved 2-year OS (24.9% vs. 10.4%) in patients with advanced biliary tract cancer.^31^ Third, the 90-day postoperative mortality was higher than most Western series. This could be partly explained by more extensive resections in patients with an R1 resection and the inclusion of patients from 25 centers rather than a small number of high-volume centers. Finally, not all patients in the palliative chemotherapy group had pathological confirmation of pCCA. These patients may have had nonmalignant disease, although this is unlikely given the negligible 3-year OS.

## Conclusions

Patients with pCCA who underwent an R1 resection had similar median OS compared with patients with localized pCCA who were treated with palliative systemic chemotherapy. Palliative systemic chemotherapy should be considered in patients with a high risk of both R1 resection and postoperative mortality.
